# Macular Ganglion Cell Inner Plexiform Layer Thickness in Glaucomatous Eyes with Localized Retinal Nerve Fiber Layer Defects

**DOI:** 10.1371/journal.pone.0160549

**Published:** 2016-08-18

**Authors:** Chunwei Zhang, Andrew J. Tatham, Ricardo Y. Abe, Na’ama Hammel, Akram Belghith, Robert N. Weinreb, Felipe A. Medeiros, Jeffrey M. Liebmann, Christopher A. Girkin, Linda M. Zangwill

**Affiliations:** 1 Department of Ophthalmology, the First Affiliated Hospital, Harbin Medical University, Harbin, China; 2 Hamilton Glaucoma Center, Department of Ophthalmology and Shiley Eye Institute, University of California San Diego, San Diego, United States of America; 3 Princess Alexandra Eye Pavilion and Department of Ophthalmology, University of Edinburgh, Edinburgh, United Kingdom; 4 Department of Ophthalmology, University of Campinas, Campinas, Brazil; 5 Harkness Eye Institute, Columbia University Medical Center, New York, New York, United States of America; 6 Department of Ophthalmology, University of Alabama, Birmingham, Alabama, United States of America; Universidade Federal do Rio de Janeiro, BRAZIL

## Abstract

**Purpose:**

To investigate macular ganglion cell–inner plexiform layer (mGCIPL) thickness in glaucomatous eyes with visible localized retinal nerve fiber layer (RNFL) defects on stereophotographs.

**Methods:**

112 healthy and 149 glaucomatous eyes from the Diagnostic Innovations in Glaucoma Study (DIGS) and the African Descent and Glaucoma Evaluation Study (ADAGES) subjects had standard automated perimetry (SAP), optical coherence tomography (OCT) imaging of the macula and optic nerve head, and stereoscopic optic disc photography. Masked observers identified localized RNFL defects by grading of stereophotographs.

**Result:**

47 eyes had visible localized RNFL defects on stereophotographs. Eyes with visible localized RNFL defects had significantly thinner mGCIPL thickness compared to healthy eyes (68.3 ± 11.4 μm versus 79.2 ± 6.6 μm respectively, P<0.001) and similar mGCIPL thickness to glaucomatous eyes without localized RNFL defects (68.6 ± 11.2 μm, P = 1.000). The average mGCIPL thickness in eyes with RNFL defects was 14% less than similarly aged healthy controls. For 29 eyes with a visible RNFL defect in just one hemiretina (superior or inferior) mGCIPL was thinnest in the same hemiretina in 26 eyes (90%). Eyes with inferior-temporal RNFL defects also had significantly thinner inferior-temporal mGCIPL (P<0.001) and inferior mGCIPL (P = 0.030) compared to glaucomatous eyes without a visible RNFL defect.

**Conclusion:**

The current study indicates that presence of a localized RNFL defect is likely to indicate significant macular damage, particularly in the region of the macular that topographically corresponds to the location of the RNFL defect.

## Introduction

Glaucoma is characterized by loss of retinal ganglion cells (RGCs) and their axons that manifests clinically as structural changes to the optic nerve head (ONH) and circumpapillary retinal nerve fiber layer (cpRNFL). [1,2] These changes are accompanied by a reduction in visual field sensitivity, which without adequate treatment, may progress to visual impairment and blindness. [3,4]

Glaucomatous damage to the ONH and cpRNFL can be observed directly using biomicroscopy or recorded using optic disc stereophotographs. Damage may also be quantified using imaging devices such as confocal scanning laser ophthalmoscopy, scanning laser polarimetry, [5,6] or optical coherence tomography (OCT). [7–9] Although imaging in glaucoma has traditionally focused on the ONH and cpRNFL, recent advances in OCT have provided the means to image the inner retina and obtain quantitative measurements of macular structures including the retinal ganglion cell layer–the location of retinal ganglion cell bodies. [10] The macular ganglion cell layer is of particular interest due to its importance for central vision and the high density of RGCs in this region. [11–13] In fact, previous studies have demonstrated the value of macular thickness measurements for glaucoma diagnosis using various parameters, including macular ganglion cell-inner plexiform layer (mGCIPL) thickness, which is the combination of the ganglion cell and inner plexiform layer. [14–19]

Glaucomatous damage may be diffuse or localized. Diffuse RGC loss is manifest by generalized loss of RNFL striations, a darkened appearance to the inner retina due to reduced reflectivity of RGC axon bundles constituting the RNFL, and increased visibility of the retinal blood vessels, which normally are embedded in the RNFL. [2,20–22] Localized damage can lead to localized RNFL defects, which are visible on stereophotographs as wedge shaped defects that become narrower towards the disc margin due to convergence of RNFL bundles.

RNFL defects are often among the earliest observable clinical signs of glaucoma. [22,23] As localized RNFL defects may be a consequence or cause of the loss of RGC axons, it is important to understand the topographic relationship between RNFL and mGCPIL thickness in eyes with localized RNFL damage. Hood et al has recently proposed a map of the topographic relationship between RNFL and GCL. In this model, the superior RNFL maps to the temporal and temporal superior GCL, while the inferior RNFL generally maps to the inferior GCL. [10,24]

The purpose this study was to examine the spatial relationship between RNFL defects and mGCIPL thickness in glaucoma patients with visible localized RNFL defects visible on optic disc stereophotographs and to compare to mGCIPL thickness measurements in healthy eyes and glaucomatous eye without visible localized RNFL defects.

## Methods

### Study Design

This was a cross-sectional study of 261 eyes of 261 patients recruited from 2 prospective longitudinal studies: the African Descent and Glaucoma Evaluation Study and the Diagnostic Innovations in Glaucoma Study. Methodological details have been described previously. [25] Patients were recruited from 3 sites; Hamilton Glaucoma Center at the Shiley Eye Institute and Department of Ophthalmology, University of California, San Diego (UCSD) (data coordinating center); the New York Eye and Ear Infirmary; and the Department of Ophthalmology, University of Alabama, Birmingham. Written informed consent was obtained from all the participants, and the institutional review boards and human subjects committees of all 3 sites prospectively approved the methods. All methods adhered to the tenets of the Declaration of Helsinki for research involving human subjects, and the study was conducted in accordance with Health Insurance Portability and Accountability Act regulations.

At each annual visit, subjects underwent a comprehensive ophthalmologic examination including review of medical history, visual acuity, slit-lamp biomicroscopy, intraocular pressure measurement, dilated fundoscopic examination and simultaneous stereoscopic optic disc photography (Kowa Nonmyd WX3D, software version VK27E, Kowa Company Ltd, Tokyo Japan) with a field angle of 34 degrees. At each biannual visit, Cirrus HDOCT (software version 6.5; Carl Zeiss Meditec, Inc. Dublin, CA), images and standard automated perimetry (SAP) using the Swedish interactive threshold algorithm (SITA standard 24–2; Carl Zeiss Meditec, Inc, Dublin, California, USA) tests were performed. Only subjects with open angles on gonioscopy were included. Subjects were excluded if at their qualifying visit, they had a best-corrected visual acuity of less than 20/40; spherical refraction outside ± 5.0 diopters, cylinder correction outside 3.0 diopters, or both; or any other ocular or systemic disease that could affect the optic nerve or the visual field. Axial length was measured using the IOL Master (Carl Zeiss Meditec, Dublin, CA) at study entry.

Eyes were classified as glaucomatous if they had repeatable (≥ 3 consecutive) abnormal SAP test results on the 24–2 program of the visual field analyzer (Humphrey Field Analyzer [HFA II-i]; Carl Zeiss Meditec, Inc., Dublin, CA) or progressive glaucomatous optic disc changes on masked examination of optic disc stereophotographs, with or without an abnormal SAP result. All visual fields were evaluated for quality by the UCSD Visual Field Assessment Center. [26] Visual fields with more than 33% fixation losses or more than 15% false-positive errors, or evidence of learning effects or other artifacts were excluded. An abnormal SAP result was defined as having a pattern standard deviation outside the 95% confidence limits or a glaucoma hemifield test outside the reference range. Healthy subjects were recruited from the general population through advertisements, primary care ophthalmic clinics and from the staff and employees of the UCSD. Healthy eyes had intraocular pressure (IOP) ≤ 21 mmHg, with no history of elevated IOP and a normal SAP result. The healthy group was matched for age with the group with visible localized RNFL defects. A sample of subjects with glaucoma without visible localized RNFL defects was selected for inclusion in the study and also matched to the age of the group with visible localized RNFL defects.

#### Stereophotograph Grading

Digital stereoscopic images were reviewed with a stereoscopic viewer (Screen-VU stereoscope; PS Manufacturing, Portland, Oregon, USA) by 2 or more experienced graders. Details of the methodology used to grade optic disc stereophotographs at the UCSD Optic Disc Reading Center have been provided elsewhere. [25,27,28] In brief, each grader was masked to the subject’s identity and to the other test results. Eyes with localized RNFL defects were identified by at least 2 graders. Localized RNFL defects were defined as defects wider than twice the width of an arteriole, extending from close to the disc margin into the peripapillary area, widening en route. (i.e., wedge shaped). [29] Slit defects narrower than the diameter of adjacent vessels were not considered as localized RNFL defects consistent with glaucoma because they are found frequently in normal eyes. [2,30]

The position of the RNFL defects at the optic disc was also assessed by graders and a clock hour numbering was adjusted to numbering of the right eye in order to allow comparison between right and left eyes, Therefore the superior-temporal region would be assigned the same clock hours in a right or left eye.

#### Optical Coherence Tomography

Cirrus HDOCT was used to acquire measurements of cpRNFL and mGCIPL thickness. Cirrus HDOCT uses a superluminescent diode scan with a center wavelength of 840 nm and an acquisition rate of 27,000 A-scans per second at an axial resolution of 5μm. The optic disc cube 200 x 200 protocol was used to acquire RNFL thickness measurements. This protocol is based on a 3-dimensional (3D) scan of a 6 x 6 mm area centered on the optic disc in which information from a 1024 (depth) x 200 x 200 point parallelepiped is collected. The RNFL thickness measurements were calculated from a 3.46-mm diameter circular scan (10,870 μm in length) automatically placed around the optic disc by the OCT software. Measurements were analyzed using 12 clock hour sectors.

The macular cube 200 x 200 protocol was used to acquire macular thickness data. This protocol is based on a 3D scan centered on the macula in which information from a 1024 (depth) x 200 x 200 point parallelepiped is collected. The ganglion cell analysis algorithm automatically segmented the GCIPL between the outer boundary of RNFL and the outer boundary of IPL based on three-dimensional data generated from the macular cube scan protocol. [31,32] The Cirrus HDOCT images were reviewed for quality by the Imaging Data Evaluation and Analysis (IDEA) Center. Images were included if the signal strength was ≥ 7, if movement artifacts and segmentation errors were absent, and there was good centering on the optic disc or fovea for the optic disc and macular cube protocols, respectively. Measurements were analyzed in 6 sectors as provided by the instrument software, superior, superior nasal, superior temporal, inferior, inferior nasal and inferior temporal. Foveal placement was manually adjusted as needed.

#### Statistical Analysis

One-way analysis of variance (ANOVA) with post-hoc analysis was done using Scheffe test to determine differences of baseline characteristics and mGCIPL measurements among the healthy group, the group with localized visible RNFL defects and the group without localized visible RNFL defects.

We also calculated the percentage of loss from mGCIPL in the glaucoma group in comparison to the healthy group. Measurements from the healthy control group were used to determine the expected mGCIPL for each sector using a linear regression model. Age and axial length were included in this model since these variables have been shown to influence mGCIPL and cpRNFL thickness measurements. [33] In healthy eyes group, age (P = 0.004) and axial length (P < 0.001) were significantly associated with mGCIPL (R^2^ = 0.23) in the multivariable model. Age (P = 0.016) and axial length (P = 0.046) were also significantly associated with cpRNFL thickness. The models had the following general form:

Expected mGCIPL thickness = constant + β1*age + β2 *axial length

Estimated mGCIPL percentage lost = [(expected mGCIPL – observed mGCIPL) /expected mGCIPL]*100

Expected cpRNFL thickness =constant+ β3*age + β4 *axial length

Estimated cpRNFL thickness percentage lost = [(expected cpRNFL – observed cpRNFL) /expected cpRNFL]*100

All statistical analyses were performed with commercially available software (STATA, version 13; Stata Corp LP, College Station, TX). The alpha level (type I error) was set at 0.05.

## Results

The study included 47 glaucomatous eyes (47 subjects) with localized RNFL defects visible on stereophotographs (RNFL defect group), 102 glaucomatous eyes (102 subjects) without visible localized RNFL defects (without localized RNFL defect group) and 112 eyes from 112 healthy subjects. There was no significant difference in age between groups (Table 1).

**Table 1:** Summary of demographic and clinical characteristics of healthy eyes compared to glaucomatous eyes with and without localized RNFL defects visible on optic disc stereo photography.

Characteristics	Healthy 112 eyes(112 subjects)	Glaucoma with localized RNFL defect 47 eyes (47subjects)	Glaucoma without localized RNFL defect 102 eyes (102 subjects)	Different among three groups (P-value*)
**Age (years)**	60.8 ± 10.1	63.5 ± 10.2	63.3 ± 10.3	0.132
**Female gender n (%)**	68 (61%)	33 (70%)	53 (52%)	0.097**
**Ancestry**				
**European**	38 (34%)	18 (38%)	40 (39%)	0.117**
**African**	72 (64%)	24 (51%)	58 (57%)	
**Other**	2 (2%)	5 (11%)	4 (4%)	
**Axial length (mm)**	23.8 ± 0.9	23.7 ± 0.8	24.0 ± 1.0	0.154
**CCT (μm)**	540.5 ± 38.5	528.5 ± 43.5	535.3 ± 38.0	0.203
**SAP MD (dB)**	0.3 ± 1.1	-5.8 ± 5.7	-6.4 ± 6.6	< 0.001†‡
**PSD (dB)**	1.6 ± 0.4	7.3 ± 4.4	6.5 ± 4.3	< 0.001†‡
**SAP VFI (%)**	99.3 ± 0.8	81.9 ± 17.9	82.5 ± 19.4	< 0.001†‡
**cpRNFL thickness (μm)**	91.6 ± 10.0	71.7 ± 9.6	72.9 ± 15.0	< 0.001†‡
**mGCIPL thickness (μm)**	79.2 ± 6.6	68.3 ± 11.4	68.6 ± 11.2	< 0.001†‡

Values are mean ± standard deviation unless specified otherwise.

* p-values are based on one-way analysis of variance with post-hoc Scheffe for two groups

**Fisher’s exact test.

Abbreviations: CCT = Central corneal thickness; SAP = standard automated perimetry; MD = mean deviation; PSD = pattern standard deviation; VFI = visual field index; cpRNFL = circumpapillary retinal nerve fiber layer; mGCIPL = macular ganglion cell layer plus inner plexiform layer.

^†^ P indicate statistically significant differences between healthy eyes and glaucomatous eyes without localized RNFL defect using one-way analysis of variance with post-hoc Scheffe (p < 0.001).

^‡^ P indicate statistically significant differences between healthy eyes and glaucomatous eyes with localized RNFL defect using one-way analysis of variance with post-hoc Scheffe (p < 0.001).

Overall glaucomatous eyes with and without localized RNFL defects visible on stereophotographs had similar disease severity, measured by SAP MD, with average MD of -5.8 ± 5.7 dB and -6.4 ± 6.6 dB respectively (Table 1). There was also no difference in average cpRNFL thickness or mGCIPL thickness between glaucomatous eyes with and without visible RNFL defects (Table 1). Average cpRNFL thicknesses were 71.7 ± 9.6, 72.9 ± 15.0 and 91.6 ± 10.0 μm in glaucomatous eyes with and without visible localized RNFL defects and healthy eyes respectively, with corresponding mGCIPL thicknesses of 68.3 ± 11.4, 68.6 ± 11.2 and 79.2 ± 6.6 μm.

**Fig 1:**
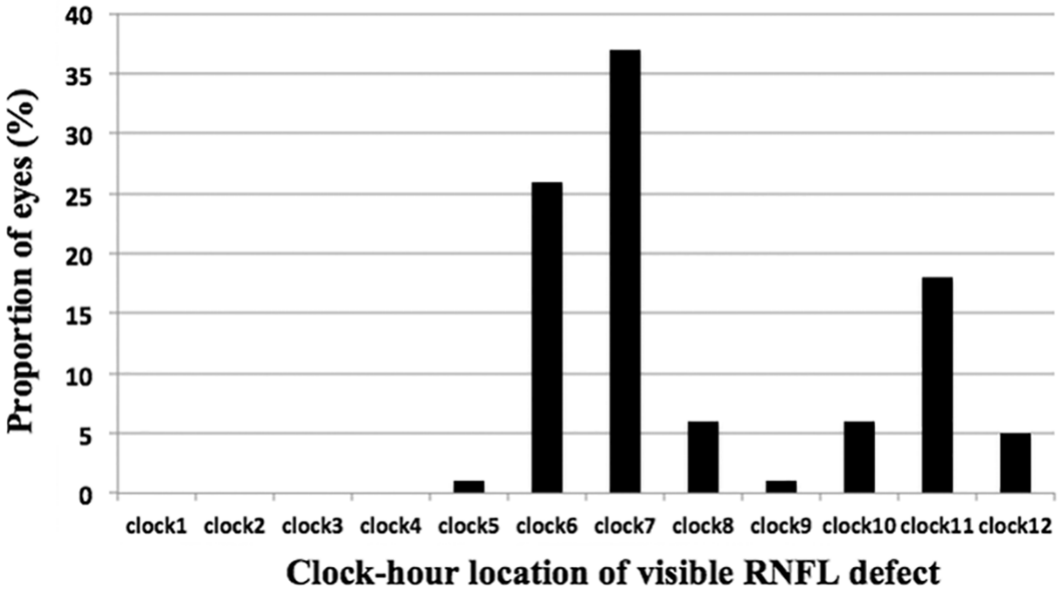
Bar graph of the distribution of eyes with visible localized retinal nerve fiber layer (RNFL) defects on optic disc stereophotography by clock-hour.

Of the 47 eyes with a visible localized RNFL defect, 29 had only one defect visible and 18 had 2 localized RNFL defects. 44 eyes had an inferior-temporal RNFL defect (clock hours 6, 7 or 8) and 21 had a superior-temporal RNFL defect (clock hours 10, 11 or 12). 26 glaucomatous eyes had an isolated inferior -temporal RNFL defect and 3 glaucomatous eyes had an isolated superior -temporal RNFL defect. The most common sectors with localized RNFL defects were 7 o’clock, 6 o’clock and 11 o’clock (Table 2 and Fig 1). The number of eyes with mGCIPL thickness outside normal limits (thinner than 1% of the Cirrus HDOCT reference database of healthy eyes, which corresponds to red sectors on the Cirrus HDOCT deviation map) in each sector is shown in Table 2.

**Table 2:** Frequency of macular ganglion cell inner plexiform layer (mGCIPL) sectors outside normal limits for glaucomatous eyes with localized retinal nerve fiber layer (RNFL) defects involving the inferior-temporal region (6, 7 or 8 o’clock) alone (A) and for glaucomatous eyes with localized RNFL defects involving the inferior-temporal and superior-temporal regions (B).

	Location of RNFL defect on optic disc photography (Clock hour)	Number of Eyes (eyes counted multiple times if more than one clock hour involved)	Number of eyes with mGCIPL sectors outside normal limits (%)*					
			ST	S	SN	IN	I	IT
**Eyes with inferior-temporal RNFL defect only (26 eyes)**	**6 o’clock**	13	5 (38%)	2 (15%)	2 (15%)	3 (23%)	8 (62%)	10 (77%)
	**7 o’clock**	18	5 (28%)	3 (17%)	4 (22%)	3 (17%)	13 (72%)	16 (89%)
	**8 o’clock**	2	1 (50%)	1 (50%)	1 (50%)	2 (100%)	2 (100%)	2 (100%)
**Eyes with both superior -temporal and inferior-temporal RNFL defects (18 eyes)**	**6 o’clock**	9	6 (67%)	4 (44%)	3 (33%)	3 (33%)	5 (56%)	6 (67%)
	**7 o’clock**	13	11 (85%)	7 (54%)	4 (31%)	5 (38%)	10 (77%)	11 (85%)
	**8 o’clock**	3	3 (100%)	3 (100%)	2 (67%)	1 (33%)	1 (33%)	1 (33%)

*Outside normal limits (<1%) compared to the Cirrus reference database of healthy eyes.

Abbreviations: mGCIPL = macular ganglion cell layer plus inner plexiform layer; ST = Superior–temporal section region to mGCIPL; S = Superior section region to mGCIPL; SN = Superior- Nasal section region to mGCIPL; IN = Inferior-Nasal section region to mGCIPL; I = Inferior section region to mGCIPL; IT = Inferior–temporal section region to mGCIPL.

40 of 47 eyes (85.1%) with a localized RNFL defect had at least one mGCIPL sector outside normal limits, compared to 13 of 112 healthy eyes (11.6%, P<0.001) and 69 of 102 glaucomatous eyes without a localized RNFL defect visible on stereophotographs (67.6%, P<0.001). There was spatial agreement between the location of visible localized RNFL defects and the location of mGCIPL sectors outside normal limits. For example, 16 of 18 eyes (89%) with an isolated RNFL defect involving 7 o’clock (inferior-temporal position) on stereophotographs had an inferior-temporal mGCIPL thickness outside normal limits, compared to 13 eyes (72%) with an abnormal inferior mGCIPL thickness, with other sectors less commonly affected (Table 2). Similar results were found for eyes with both inferior-temporal and superior-temporal RNFL defects (Table 2). 12 of 15 eyes (80%) with localized RNFL defects visible at 11 o’clock (superior-temporal position) had a superior-temporal mGCIPL thickness outside normal limits–with superior and superior-nasal mGCIPL thickness also affected. The average mGCIPL thickness was thinnest in the hemiretina corresponding to the visible RNFL defect in 26 of 29 eyes (90%) with one localized RNFL defect.

Table 3 shows mGCIPL thicknesses in each sector for eyes with inferior-temporal RNFL defects compared to eyes without visible localized RNFL defects. Although overall glaucomatous eyes included in this study had similar disease severity, eyes with localized RNFL loss visible on stereophotographs had evidence of greater localized mGCIPL thickness loss at the expected location based on the Hood et al map. [10] For example, eyes with inferior-temporal RNFL defects had significantly thinner inferior and inferior-temporal mGCIPL thickness than glaucomatous eyes without localized RNFL defects (61.7 ± 12.4 and 66.9 ± 12.8, P = 0.030 and 59.3 ± 11.0 and 67.8 ± 12.1μm, P<0.001 respectively, Table 3). Eyes with inferior or inferior-temporal RNFL defects had significantly thinner mGCIPL compared to healthy eyes in all sectors (Table 3). In our study, there were 21 eyes with superior-temporal RNFL defects but only 3 eyes with isolated superior-temporal RNFL defects. We also found that in the 21 eyes with superior-temporal RNFL defects (10,11 and 12 o’clock) tended to have thinner superior-temporal mGCIPL compared to glaucomatous eyes without localized RNFL defects, but the difference did not reach statistical significance (62.8 ± 11.1μm versus 67.6 ± 11.2μm, P = 0.104). The result is likely in part due to insufficient sample size to examine this.

**Table 3:** Summary of macular ganglion cell inner plexiform layer (mGCIPL) thickness in healthy eyes compared to glaucomatous eyes without visible localized RNFL defects and with visible localized RNFL defects involving inferior and inferior-temporal region (6, 7 or 8 o’clock).

Characteristics	112 eyes (112 subjects)	Glaucoma with localized RNFL defect at 6, 7, or 8 o’clock 44 eyes (44 subjects)	Glaucoma without localized RNFL defect 102 eyes(102 subjects)	Glaucoma with localized RNFL defect at 6, 7, or 8 o’clock Vs. healthy (P-value)*	Glaucoma with localized RNFL defect at 6, 7, or 8 o’clock Vs. Glaucoma without localized RNFL defect (P-value)*
**mGCIPL thickness- total (μm)**	79.2 ± 6.6	67.9 ± 11.4	68.6 ± 11.2	< 0.001	0.929
**mGCIPL thickness–lower half (μm)**	79.0 ± 6.6	64.4 ± 11.0	68.0 ± 12.2	< 0.001	0.148
**Inferior- Temporal mGCIPL thickness (μm)**	79.8 ± 6.5	59.3 ± 11.0	67.8 ± 12.1	< 0.001	< 0.001
**Inferior mGCIPL thickness (μm)**	78.2 ± 7.2	61.7 ± 12.4	66.9 ± 12.8	< 0.001	0.030
**Inferior- Nasal mGCIPL thickness (μm)**	79.0 ± 7.3	72.3 ± 12.5	69.2 ± 13.0	0.002	0.306
**Superior- Temporal mGCIPL thickness (μm)**	78.2 ± 6.9	67.3 ± 14.0	67.6 ± 11.2	< 0.001	0.979
**Superior mGCIPL thickness (μm)**	79.5 ± 7.2	71.5 ± 15.5	68.9 ± 12.5	< 0.001	0.418
**Superior- Nasal mGCIPL thickness (μm)**	80.3 ± 7.7	75.4 ± 15.6	71.1 ± 12.0	0.051	0.106

Values are mean ± standard deviation and

* One-way analysis of variance with post-hoc Scheffe for two groups.

Abbreviations: MD = mean deviation; PSD = pattern standard deviation; SAP = standard automated perimetry; MD = mean deviation; mGCIPL = macular ganglion cell layer plus inner plexiform layer.

**Fig 2:**
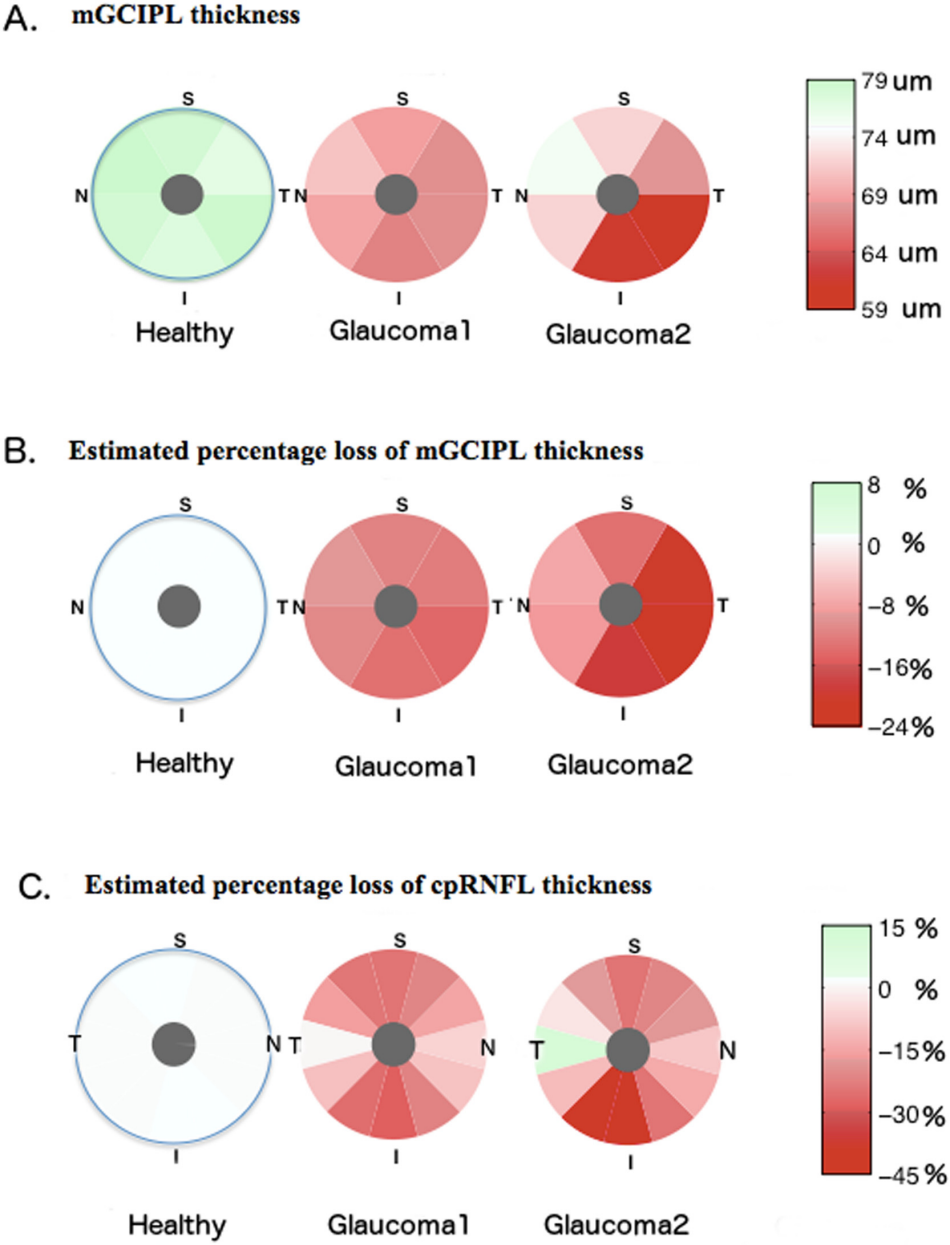
Pie charts showing the regional distribution of macular ganglion cell layer plus inner plexiform layer (mGCIPL) thickness (A), estimated percentage loss of mGCIPL thickness (B) and estimated percentage loss of circumpapillary retinal nerve fiber layer (cpRNFL) thickness (C) in 112 healthy eyes, 102 glaucomatous eyes without visible retinal nerve fiber layer (RNFL) defects (Glaucoma 1) and 44 glaucomatous eyes with a visible inferior -temporal RNFL defect (Glaucoma 2).

**Fig 3:**
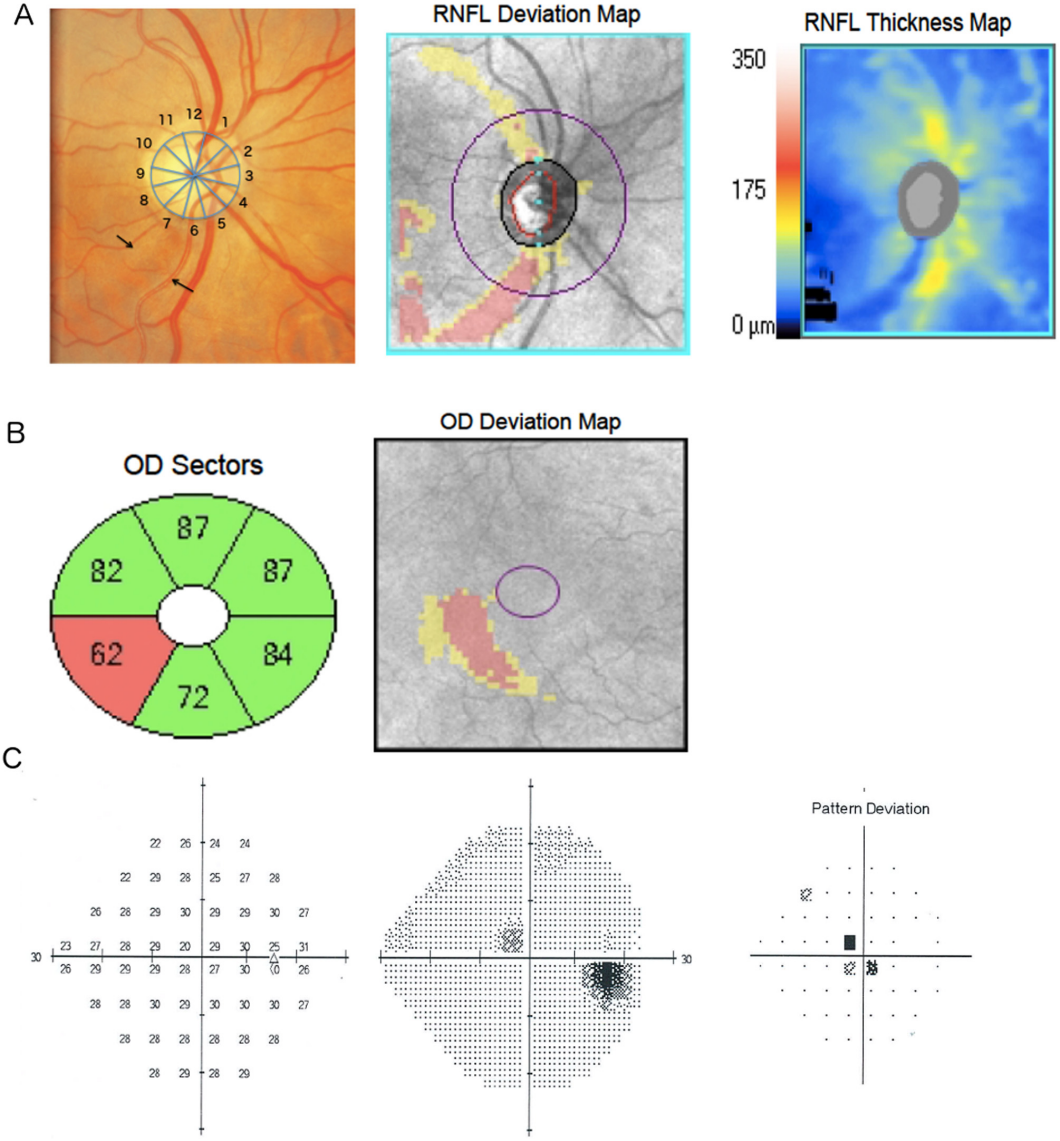
Example of a glaucomatous eye with a localized retinal nerve fiber layer (RNFL) defect at 6 and 7 o’clock (arrows) visible on photography and optical coherence tomography RNFL deviation and thickness maps (A). There is corresponding localized inferior-temporal macular ganglion cell-inner plexiform layer thinning (B) and a paracentral defect visible on standard automated perimetry (SAP). The average percentage estimated mGCIPL loss for this patient was 6.5%, with a 17.9% estimated loss in the inferior-temporal mGCIPL sector.

Fig 2 shows the regional distribution of mGCIPL thickness, and estimated percentage loss of mGCIPL and cpRNFL thickness in glaucomatous eyes with inferior-temporal defects compared to glaucomatous eyes without a visible RNFL defect and healthy eyes. Examples of eyes with localized RNFL defects visible on stereophotographs included in the study are shown in Fig 3.

## Discussion

Recently, there has been renewed emphasis on the importance of evaluating the macula in glaucoma diagnosis and management. [16] Glaucomatous damage to the macula is common, can occur early in the disease, and can be missed and/or underestimated with standard visual field tests. [10] The present study shows that patients with localized RNFL defects visible on stereophotographs may have substantial macular damage, with potentially important implications for visual function.

Patients with localized RNFL defects visible on stereophotographs had an average mGCIPL thickness of 68.3 ± 11.4 μm, which was 14% less than healthy subjects of a similar age. Therefore, although RNFL defects are considered one of the earliest changes of glaucoma and often precede noticeable change of the ONH and visual field, the current study suggests they may be associated with detectable mGCIPL losses. [22,23,34] In eyes with localized RNFL defects, mGCIPL thickness ranged from 28 to 88 μm, reflecting an average estimated loss of 11% to 63%. Average mGCIPL thickness was also reduced in the expected sectors that correspond to visible RNFL defects. For example, in healthy eyes the inferior-temporal mGCIPL average thickness was 79.8 μm, while in eyes with inferior-temporal RNFL defects, the average inferior-temporal mGCIPL thickness was 59.3 μm, which was 26% less than that expected in a healthy eye.

Our results are generally consistent with Hood et al’s map of the relationship between the location of RNFL and macular damage. They also are consistent with the idea that there is a macular vulnerability zone that is particularly susceptible to damage. [24] In eyes with inferior-temporal RNFL defects, the GCIPL tended to be thinner in the inferior and inferior-temporal region. Further, in eyes with RNFL superior damage, the GCIPL was thinner in the superior and superior-temporal macular region. Specifically, most eyes with inferior-temporal RNFL defects located at 6 o-clock and 7 o-clock detected on stereophotographs had inferior or inferior- temporal thickness outside normal limits. Similarly, most eyes with superior RNFL damage at 10 o-clock and 11 o-clock positions have damage in the superior-temporal macular region. Moreover, few eyes with localized superior RNFL defects at 12 o-clock had GCIPL damage outside normal limits in the area measured, as the nerve fibers tend to project superiorly outside of the standard GCIPL measurement area.

To the best of our knowledge, there are no histological studies specifically examining macula changes in eyes with localized RNFL defects based on stereophotographs, however a previous histological study has shown that localized RNFL defects were only visible on photographs once there was a 28 to 45% reduction in RNFL. [2] We found average cpRNFL thickness in eyes with localized RNFL defects was 71.7 ± 9.6μm, or 22% less than healthy subjects. Compared to RNFL thickness in healthy eyes, RNFL loss in sectors corresponding to the RNFL defect visible on optic disc stereophotographs averaged almost 37%. Tatham and colleagues recently estimated that patients with localized RNFL defects are associated with a loss of 32% of retinal ganglion cells in affected sectors. [35] Recently, Kim and colleagues have produced a mGCIPL deviation frequency map showing the topographic relationship between mGCIPL defects and cpRNFL defects on OCT. [36] The same group have also demonstrated mGCIPL thickness to have similar ability to detect glaucomatous eyes with localized RNFL defects compared to cpRNFL measurements. [37] These studies have enhanced our understanding of the relationship between cpRNFL and mGCIPL loss in glaucoma but longitudinal studies are needed to examine the temporal relationship between changes in these structures. Future work in this area may lead to improved understanding of the mechanisms of glaucomatous damage and identify methods to better detect glaucoma.

Localized RNFL defects are areas of abnormality within areas of relatively preserved RNFL [38], and therefore, the visibility of a localized RNFL defect depends on the thickness and arrangement of the RNFL in neighboring regions. However, eyes with localized RNFL loss often also have diffuse loss, and localized RNFL defects tend to progress by deepening and widening to become diffuse defects. [30,39] The diffuse loss on the cpRNFL thickness could also reflect a diffuse macular damage. For example, from Tables 2 and 3 it is apparent that although eyes with inferior or inferior-temporal RNFL defects had maximal mGCIPL loss in the inferior-temporal sectors compared to healthy subjects, the mGCIPL was also thinner in all sectors. The mGCIPL was thinner, however, in sectors that topographically map to the location of the RNFL defects compared to sectors which do not map directly to the RNFL defect location, and compared to sectors of healthy eyes (Table 3).

The study has some limitations. Identification of localized RNFL defects was based on stereophotographs rather than HDOCT, however this was integral to the study as we wished to ascertain mGCIPL losses associated with clinically visible RNFL defects and hypothesized that visible RNFL defects would be associated with large mGCIPL losses. Nevertheless, it is possible that some glaucomatous eyes may have had localized RNFL defects that were not detected. Efforts were taken to minimize this possibility by having 2 masked graders assess the stereophotographs. It is also possible that differences in alignment between stereophotographs and HDOCT may have introduced error in our analysis of spatial agreement between RNFL defects and mGCIPL loss. However, this would not have affected the average measurements, moreover all images were reviewed for quality and we examined the spatial relationship between RNFL defects and mGCIPL using large sectors that would be less subject to differences in alignment. Future studies should also investigate the longitudinal relationship between mGCIPL thickness in eyes with localized RNFL defects.

Despite limitations, identification of RNFL defects on stereophotographs or slit lamp biomicroscopy remains an important component of the clinical examination in those with glaucoma or suspected glaucoma. The current study indicates that the presence of a localized RNFL defect is also likely to indicate significant macular damage, particularly in the region of the macular that topographically corresponds to the location of the RNFL defect. Although visible localized RNFL defects are considered an early sign of glaucoma, they are in fact associated with significant glaucomatous macular neural losses.

## Supporting Information

S1 Table Dataset. (XLSX)
